# Ceramide-1-phosphate is a regulator of Golgi structure and is co-opted by the obligate intracellular bacterial pathogen *Anaplasma phagocytophilum*

**DOI:** 10.1128/mbio.00299-24

**Published:** 2024-02-28

**Authors:** Curtis B. Read, Anika N. Ali, Daniel J. Stephenson, H. Patrick Macknight, Kenneth D. Maus, Chelsea L. Cockburn, Minjung Kim, Xiujie Xie, Jason A. Carlyon, Charles E. Chalfant

**Affiliations:** 1 Department of Microbiology and Immunology, Virginia Commonwealth University Medical Center, School of Medicine, Richmond, Virginia, USA; 2 Department of Cell Biology, Microbiology, and Molecular Biology, University of South Florida, Tampa, Florida, USA; 3 Division of Hematology & Oncology, Department of Medicine, University of Virginia, Charlottesville, Virginia, USA; 4 Department of Cell Biology, University of Virginia, Charlottesville, Virginia, USA; 5 Program in Cancer Biology, University of Virginia Cancer Center, Charlottesville, Virginia, USA; 6 Research Service, Richmond Veterans Administration Medical Center, Richmond, Virginia, USA; Yale University School of Medicine, New Haven, Connecticut, USA

**Keywords:** ceramide-1-phosphate, Golgi, GRASP, GoRASP, ceramide kinase, ceramide-1-phosphate transport protein, *Anaplasma phagocytophilum*, intracellular bacterial pathogen, host-pathogen interaction

## Abstract

Many intracellular pathogens structurally disrupt the Golgi apparatus as an evolutionarily conserved promicrobial strategy. Yet, the host factors and signaling processes involved are often poorly understood, particularly for *Anaplasma phagocytophilum*, the agent of human granulocytic anaplasmosis. We found that *A. phagocytophilum* elevated cellular levels of the bioactive sphingolipid, ceramide-1-phosphate (C1P), to promote Golgi fragmentation that enables bacterial proliferation, conversion from its non-infectious to infectious form, and productive infection. *A. phagocytophilum* poorly infected mice deficient in ceramide kinase, the Golgi-localized enzyme responsible for C1P biosynthesis. C1P regulated Golgi morphology via activation of a PKCα/Cdc42/JNK signaling axis that culminates in phosphorylation of Golgi structural proteins, GRASP55 and GRASP65. siRNA-mediated depletion of Cdc42 blocked *A. phagocytophilum* from altering Golgi morphology, which impaired anterograde trafficking of *trans*-Golgi vesicles into and maturation of the pathogen-occupied vacuole. Cells overexpressing phosphorylation-resistant versions of GRASP55 and GRASP65 presented with suppressed C1P- and *A. phagocytophilum*-induced Golgi fragmentation and poorly supported infection by the bacterium. By studying *A. phagocytophilum*, we identify C1P as a regulator of Golgi structure and a host factor that is relevant to disease progression associated with Golgi fragmentation.

**IMPORTANCE:**

Ceramide-1-phosphate (C1P), a bioactive sphingolipid that regulates diverse processes vital to mammalian physiology, is linked to disease states such as cancer, inflammation, and wound healing. By studying the obligate intracellular bacterium *Anaplasma phagocytophilum*, we discovered that C1P is a major regulator of Golgi morphology. *A. phagocytophilum* elevated C1P levels to induce signaling events that promote Golgi fragmentation and increase vesicular traffic into the pathogen-occupied vacuole that the bacterium parasitizes. As several intracellular microbial pathogens destabilize the Golgi to drive their infection cycles and changes in Golgi morphology is also linked to cancer and neurodegenerative disorder progression, this study identifies C1P as a potential broad-spectrum therapeutic target for infectious and non-infectious diseases.

## INTRODUCTION

**Fig 1:**
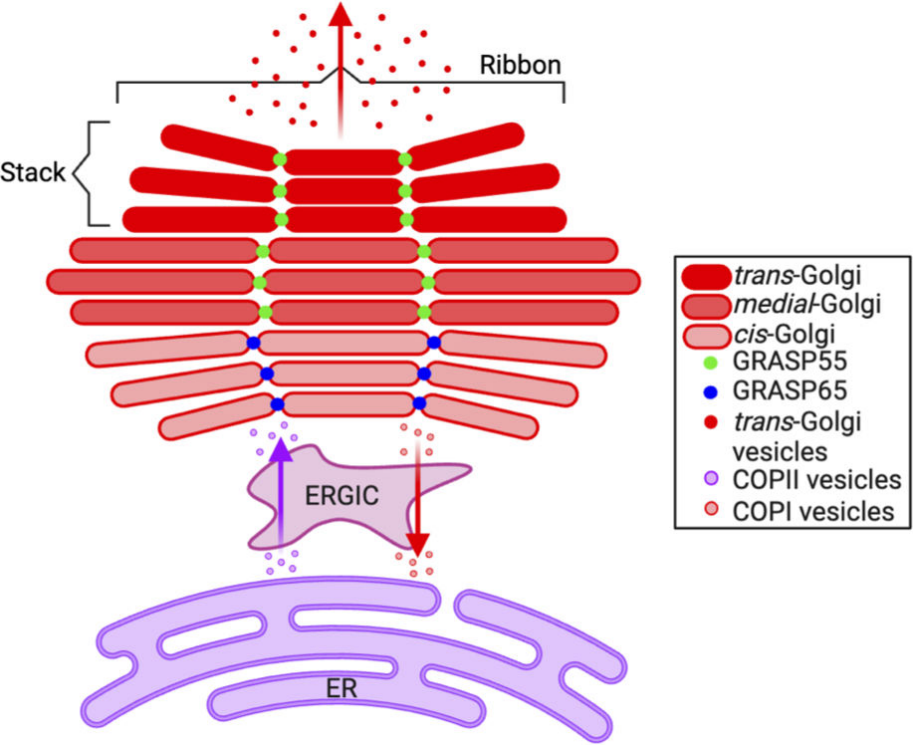
Golgi trafficking and the contributions of GRASP55 and GRASP65 to ribbon linking. The Golgi consists of closely opposed cisternae that are aligned in parallel into stacks and laterally connected to form a continuous ribbon. GRASP65, which is concentrated in the *cis*-Golgi, and GRASP55, which is present in the *medial*- and *trans*-Golgi, link the stacks into the ribbon. In anterograde trafficking, proteins and lipids from the ER are packaged into COPII-coated vesicles that are delivered through the ERGIC to the *cis*-Golgi cisternae and migrate through the stack to the TGN for export to other organelles or out of the cell. In retrograde trafficking, misfolded proteins are transported from the Golgi through the ERGIC to the ER via COPI vesicles.

The Golgi apparatus, the central hub of the secretory pathway, consists of several closely opposed cisternae that are aligned in parallel into stacks and laterally connected to form a continuous ribbon. In anterograde trafficking, newly synthesized proteins and lipids from the endoplasmic reticulum (ER) are packaged into coat protein complex II (COPII)-coated vesicles that are delivered through the ER-Golgi intermediate compartment (ERGIC) to the *cis*-Golgi cisternae and migrate through the stack to the *trans*-Golgi network (TGN) for delivery to other organelles or out of the cell (Fig. 1). In retrograde trafficking, COPI vesicles transport misfolded proteins from the Golgi through the ERGIC to the ER (1, 2). Golgi matrix proteins, including Golgi reassembly stacking proteins (GRASPs) and golgins, maintain Golgi structure. Golgins tether vesicles to cisternal membranes (2). Two recent studies confirmed that, contrary to their name, GRASPs do not hold the cisternae into stacks but instead link the stacks into the Golgi ribbon (3, 4). GRASP65 (GRASP of 65 kDa), concentrated in the *cis*-Golgi, and GRASP55, present in the *medial*- and *trans*-Golgi, perform non-redundant roles in ribbon linking (5, 6) (Fig. 1). Both also critically influence Golgi vesicle transport (3, 7).

The Golgi is a dynamic organelle that undergoes morphological changes under certain physiologic conditions (2). Knockout or acute degradation of GRASP55 and GRASP65 leads to ribbon uncoupling (3, 4, 7); and this structural change has been proposed to indirectly result from the imbalance of cargo transport caused by GRASP depletion (3). GRASP phosphorylation also promotes loss of ribbon integrity. Cyclin-dependent kinase 1 phosphorylation of GRASP55 and GRASP65 at the onset of mitosis leads to ribbon unlinking (2, 8–10). c-Jun N terminal kinase (JNK) and protein kinase Cα (PKCα)) phosphorylation of GRASP65 and GRASP55, respectively, also induce ribbon disruption. Cell division control protein 42 (Cdc42) modulates Golgi morphology and Golgi-to-ER retrograde trafficking upstream of JNK (11–13). In its active GTP-bound state, Cdc42 associates with cellular membranes and binds COPI, which culminates in retrograde traffic disruption and Golgi destabilization (14, 15). GTP-bound Cdc42 also accelerates Golgi anterograde trafficking (16). Moreover, PKCα prompts Cdc42 membrane localization (17, 18), indicating that, in addition to directly inducing Golgi disruption by phosphorylating GRASP55 (19), it indirectly modulates Golgi stability through Cdc42. Golgi morphology is also altered under certain pathologic conditions including inflammation, cancer, and neurodegeneration (1, 20). Importantly, Golgi destabilization does not impair transport function but rather yields more membrane surfaces for vesicle budding and thereby accelerates anterograde transport (2, 21), a phenomenon that ties in with the influential roles of GRASP55 and GRASP65 on vesicle transport and Golgi structural integrity (3, 4, 7).

Perturbation of Golgi structure is a driver of infectious disease pathogenesis. Numerous intracellular bacterial, viral, and protozoan pathogens induce Golgi destabilization as an evolutionarily conserved strategy to parasitize nutrients and organelle membranes, replicate, and subvert immune defenses (22–40). One such microbe is *Anaplasma phagocytophilum*, a tick-transmitted obligate intracellular bacterium and the cause of human granulocytic anaplasmosis (HGA). This acute non-specific febrile illness can result in shock, sepsis, disseminated intravascular coagulation, inflammatory syndromes, renal failure, hemorrhages, rhabdomyolysis, and death (41). *A. phagocytophilum* primarily invades neutrophils to reside in a host cell-derived multivesicular body that it modifies (41, 42). In the ApV (*A. phagocytophilum*-occupied vacuole), the bacterium undergoes biphasic development whereby shortly after invasion its infectious dense-cored (DC) form converts to the non-infectious reticulate cell (RC) morphotype that replicates to fill the lumen (43). Around 24 h post-infection (hpi), the RCs transition to DCs, which are released by MVB exocytosis to infect other cells (43, 44). Progression of this infection cycle depends on *A. phagocytophilum* parasitizing sphingolipid-rich TGN-derived vesicles that are anterograde trafficked into the ApV lumen per normal MVB biogenesis (25, 45). TGN-derived vesicle delivery into the ApV is upregulated during RC replication, is linked to bacterial uptake of sphingomyelin and ceramide, and is important for RC-to-DC conversion and infectious progeny release (25).

Ceramide-1-phosphate (C1P) is a bioactive sphingolipid that plays critical roles in health and disease including cell survival, apoptosis, migration, autophagy, and inflammation (46–64). Given the diverse processes that it regulates, C1P would be an excellent target for intracellular microbes to modulate for converting their host cells into permissive niches. Yet, such a role for C1P in the molecular pathogenesis of any infection has not been demonstrated. Three lines of evidence implicate C1P as a keystone factor for Golgi fragmentation and TGN parasitism during *A. phagocytophilum* infection. First, acid sphingomyelinase (aSMase), an MVB resident enzyme that converts sphingomyelin to ceramide (65), is critical for ApV expansion and maturation, RC-to-DC conversion, DC release from host cells, and is essential for *A. phagocytophilum* productive infection in mice (66, 67). Second, ceramide kinase (CERK), which generates C1P via direct phosphorylation of aSMase-derived ceramide, localizes to the TGN (56). Third, C1P is linked to the activation of PKCα and JNK that phosphorylate GRASP55 and GRASP65 to induce Golgi fragmentation and accelerate TGN anterograde traffic (19, 62, 68, 69).

In this study, we investigated the hypothesis that increases in C1P levels induced by *A. phagocytophilum* disrupt Golgi structure to bolster infection. Elevated C1P levels were found to benefit the bacterium during infection of myeloid cells and mice, and the sphingolipid disrupts Golgi structure by promoting Cdc42 membrane-binding, PKCα phosphorylation of GRASP55, and JNK phosphorylation of GRASP65. Cells overexpressing phosphorylation-resistant versions of GRASP55 and GRASP65 are inhibited for both C1P- and *A. phagocytophilum*-induced Golgi fragmentation and poorly support *A. phagocytophilum* infection. Overall, C1P is a major regulator of Golgi morphology that can be exploited for pathogenic endosymbiont success.

## RESULTS

### C1P regulates Golgi morphological changes induced by *A. phagocytophilum* infection

**Fig 2:**
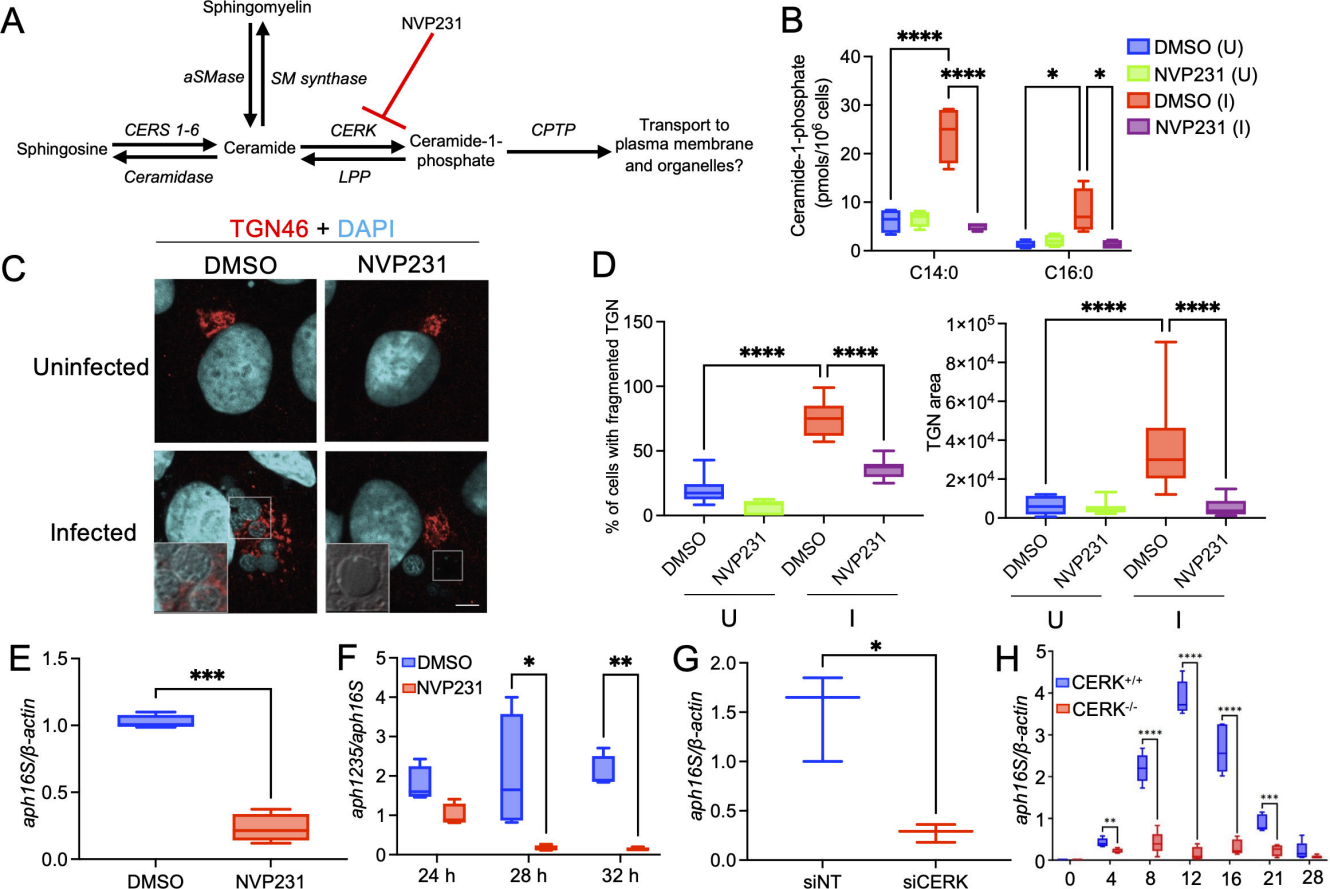
CERK-derived C1P regulates Golgi morphological changes induced by and is critical for *A. phagocytophilum* infection. (**A**) Schematic of sphingolipid metabolism. Ceramide, center of sphingolipid metabolism, is generated by either *de novo* synthesis by ceramide synthases (CERS) or by catabolism of sphingomyelin (SM) via a sphingomyelinase (SMase) [acid sphingomyelinase (aSMase) is depicted]. Ceramide is phosphorylated by CERK to generate C1P, which is transported by CPTP to the plasma membrane and other organelles, where it is catabolized by lipid phosphatases (LPP). (**B**) CERK-derived C1P is induced by *A. phagocytophilum* infection. RF/6A cells were pretreated with either NVP231 (400 nM) or control (0.001% DMSO) for 1 h, followed by incubation with *A. phagocytophilum* DC organisms (I) or left uninfected (U). At 24 h, C1P levels were analyzed using UPLC-ESI-MS/MS with the two main chain lengths of C1P depicted. One-way ANOVA with Tukey’s post hoc test was used to test for significant differences in D-e-C_14:0_ C1P and D-e-C_16:0_ C1P levels among the conditions. (**C and D**) CERK-derived C1P is required for *A. phagocytophilum*-induced Golgi fragmentation. RF/6A cells were treated as in (**B**). At 24 h post-infection, the cells were fixed, immunolabeled with TGN46 antibody (Alexa Fluor 594 secondary, red), stained with DAPI (blue) to visualize host cell nuclei and bacterial nucleoids, and examined using laser scanning confocal microscopy (LSCM). Merged fluorescence images are shown (**C**). The regions that are denoted by hatched lined boxes are magnified in the insets that are demarcated by solid lined boxes. Scale bar, 10 µm. (**D**) Fluorescence micrographs were examined to determine the mean (±SD) percentage of cells with dispersed Golgi and mean (±SD) Golgi area. Data are representative of four independent experiments in which 50 cells were examined per condition each time. Statistical analysis was performed using one-way ANOVA with Tukey’s post hoc test. (**E and F**) CERK inhibition inhibits *A. phagocytophilum* infection and RC-to-DC conversion. RF/6A cells were treated as in (**B**). qRT-PCR and the 2^−ΔΔCT^ method were used to measure the bacterial load as relative *A. phagocytophilum* 16S rRNA gene (*aph16S*)-to-human *β-actin* expression at 24 h post-infection (**E**) and relative *aph1235*-to-*aph16s* expression at 24, 28, and 32 h post-infection (**F**). Data are indicative of four separate experiments. Statistical analysis was performed using an unpaired, two-tailed *t* test with Welch’s correction for panel (E) and using one-way ANOVA with Tukey’s post-hoc test for Panel F. (**G**) siRNA downregulation of CERK abrogates *A. phagocytophilum* infection. RF/6A cells were transfected with CERK siRNA (siCERK) or non-targeting control siRNA (siNT), followed by incubation with *A. phagocytophilum* DC organisms 48 h later. At 24 h post-infection, the bacterial load was determined using qRT-PCR. Data are representative of three independent experiments. Statistical analysis was performed using an unpaired, two-tailed *t* test with Welch’s correction. (**H**) *A. phagocytophilum* fails to productively infect CERK^−/−^ mice. Wild-type (CERK^+/+^) and CERK^−/−^ mice were injected intraperitoneally with 1 × 10^8^ *A. phagocytophilum* DC organisms. Peripheral blood samples collected on day 0 (prior to infection) and the indicated days post-infection were analyzed by qPCR and the 2^−ΔΔCT^ method to measure the relative *A. phagocytophilum aph16S* gene to murine DNA levels. Data are representative of two independent experiments each conducted with four to five male and female mice per group (total of 9–10 mice per group). Statistical analysis was performed using repeated measures ANOVA *P < 0.05, **P < 0.01, ***P < 0.001, and ****P < 0.0001.

Because ceramide generated by aSMase is a precursor of CERK-derived C1P (Fig. 2A), CERK is localized in the TGN (56), delivery of TGN-derived vesicles enriched in ceramide and sphingomyelin into the ApV is critical for *A. phagocytophilum* infection cycle progression (25), and disrupting ceramide generation by inhibiting aSMase halts the infection cycle (66, 67), C1P levels were examined in infected versus uninfected cells using ultra high performance-liquid chromatography-electrospray ionization-tandem mass spectrometry (UPLC-ESI-MS/MS). Human promyelocytic HL-60 cells and primate RF/6A choroidal endothelial cells are well-established models for studying *A. phagocytophilum*-host interactions (25, 43, 44, 66, 70–73). Additionally, RF/6A clls are large and flat, which make them excellent host cells for microscopically imaging the ApV (25, 42, 44, 66, 71, 72, 74–79). *A. phagocytophilum* induced significant increases in the levels of D-e-C_14:0_ C1P and D-e-C_16:0_ C1P in both cell types (Fig. 2B; Fig. S1A). This phenomenon was blocked by the CERK inhibitor, NVP231. NVP231-mediated C1P depletion also inhibited TGN fragmentation and TGN area increase in *A. phagocytophilum* infected cells (Fig. 2C and D; Fig. S1B and C). Thus, *A. phagocytophilum* perturbs Golgi structure by a C1P-regulated pathway.

### CERK-derived C1P is critical for *A. phagocytophilum* intracellular replication, infection cycle progression, and productive infection in mice

To determine if CERK-derived C1P is required for *A. phagocytophilum* infection, HL-60 and RF/6A cells infected in the presence of NVP231 were examined at 24 hpi by qPCR. Decreases in the bacterial DNA load of approximately 98% and 50% were observed in NVP231-treated RF/6A and HL-60 cells, respectively (Fig. 2E; Fig. S1D). To assess the importance of CERK-derived C1P for the pathogen’s conversion from the replicative RC morphotype to the infectious DC form, the experiment was repeated to assess the expression of *aph1235*, which encodes a DC-specific marker that is induced when *A. phagocytophilum* undergoes RC-to-DC conversion between 24 and 32 h (73). Whereas *aph1235* expression increased over the time course for control cells, its levels were pronouncedly reduced in NVP231-treated cells (Fig. 2F; Fig. S1E). Next, CERK was downregulated in RF/6A cells by siRNA, followed by incubation with DC organisms. CERK downregulation induced a several-fold reduction in the bacterial DNA load (Fig. 2G). To define the relevance of CERK to *A. phagocytophilum* infection *in vivo*, CERK^−/−^ or wild-type mice were inoculated with DC organisms after which the bacterial load in the peripheral blood was determined by qPCR. In wild-type mice, bacterial load peaked by day 12, followed by a gradual decline to nearly undetectable levels by day 28 (Fig. 2H). *A. phagocytophilum* DNA was barely detectable at all time points in CERK^−/−^ mice.

**Fig 3:**
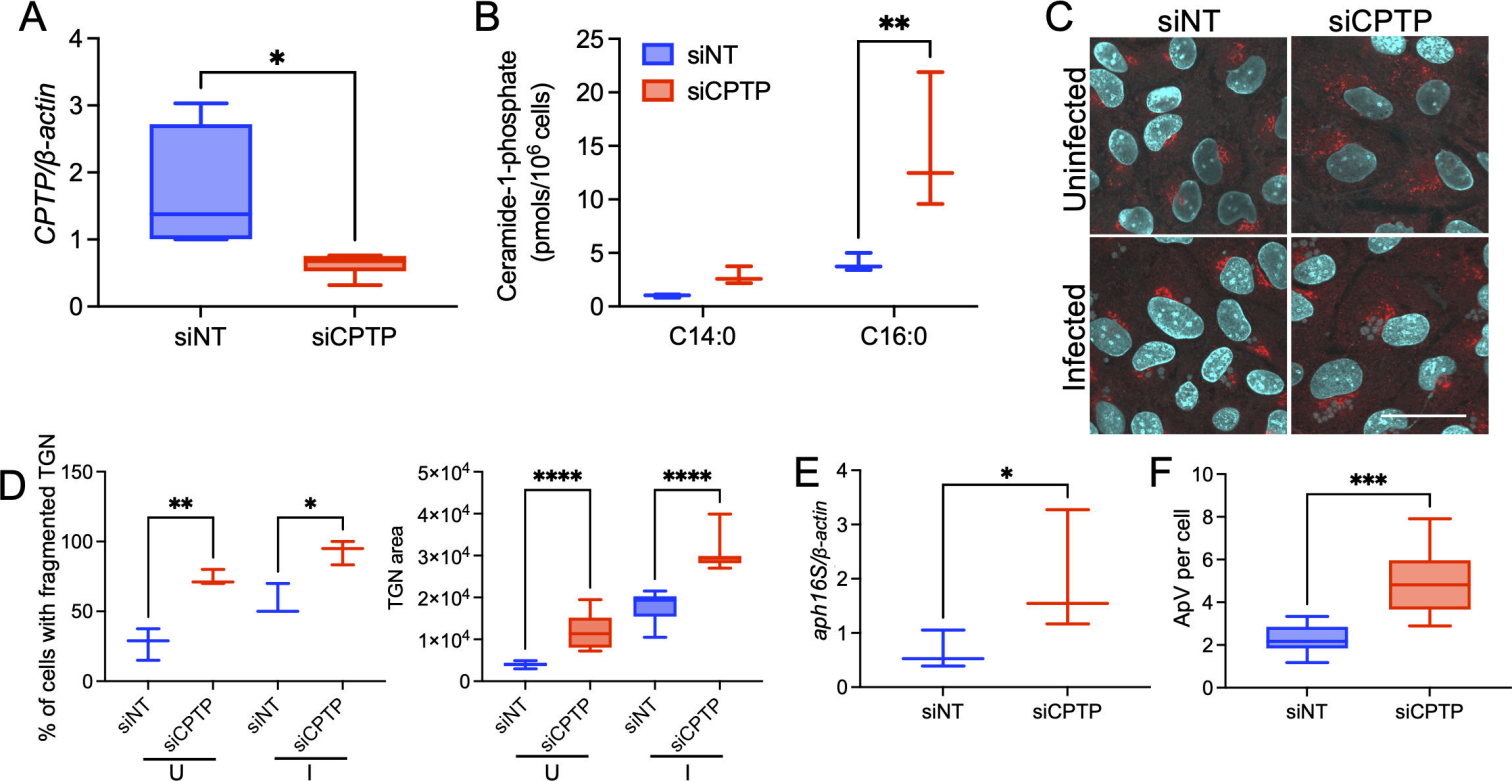
Elevating C1P levels via CPTP siRNA-mediated downregulation enhances Golgi fragmentation and benefits *A. phagocytophilum* infection. (**A and B**) CPTP downregulation raises C1P levels. RF/6A cells were transfected with CPTP siRNA (siCPTP) or siNT. At 48 h, the cells were incubated with *A. phagocytophilum* DC organisms. At 24 h post-infection, qRT-PCR and the 2^−ΔΔCT^ method were used to assess relative *CPTP*-to-*β-actin* expression (**A**), while C1P levels were measured using UPLC-ESI-MS/MS (**B**). Data in (**A**) and (**B**) are representative of four and three independent experiments, respectively. Statistical analysis was performed using an unpaired, two-tailed *t* test with Welch’s correction for panel (A) and one-way ANOVA with Tukey’s multiple comparisons test for panel (B). (**C and D**) Elevating C1P levels enhances Golgi dispersal in uninfected and *A. phagocytophilum* infected cells. RF/6A cells were treated with siNT or siCPTP for 48 h were incubated with *A. phagocytophilum* DC organisms (Infected, I) or not (Uninfected, U). At 24 h post-infection, the cells were fixed, immunolabeled with antibody against TGN46 (Alexa Fluor 594 secondary, red), stained with DAPI (blue) to visualize host cell nuclei and bacterial nucleoids, and examined by LSCM. Merged fluorescence images are shown (**C**). Scale bar, 10 µm. (**D**) Fluorescence micrographs were examined to determine the mean (±SD) percentage of cells with fragmented Golgi and mean (±SD) Golgi area. Data are representative of four independent experiments in which 50 cells were examined per condition each time. Statistical analysis was performed using one-way ANOVA with Tukey’s post hoc test. (**E and F**) CPTP downregulation increases the *A. phagocytophilum* load and the number of ApVs per cell. RF/6A cells were treated as in (**A**). (**E**) At 24 h post-infection, qRT-PCR and the 2^−ΔΔCT^ method were used to measure the bacterial load as relative *A. phagocytophilum* 16S rRNA gene (*aph16S*)-to-*β-actin* expression. (**F**) Fluorescence micrographs were examined to determine the mean (±SD) number of ApVs per cell. Data are representative of at least three independent experiments. Statistical analyses were performed using an unpaired, two-tailed *t* test with Welch’s correction. *P < 0.05; **P < 0.01; ***P < 0.001; and ****P < 0.0001.

To further validate the importance of C1P to *A. phagocytophilum* infection, we investigated the effects of increasing C1P levels on bacterial*-*induced Golgi fragmentation, the *Anaplasma* load, and ApV expansion. C1P levels were elevated by siRNA-mediated downregulation of CPTP as confirmed by qRT-PCR (Fig. 3A) and UPLC-ESI-MS/MS analysis (Fig. 3B). Elevating C1P levels enhanced *A. phagocytophilum-*induced Golgi fragmentation (Fig. 3C and D), and concomitantly increased the bacterial DNA load and ApV number per cell (Fig. 3E and F). Collectively, these data demonstrate that CERK-derived C1P is critical for *A. phagocytophilum* to promote Golgi fragmentation, which, in turn, is important for it to replicate, progress through its infection cycle, and productively infect mammalian hosts.

### CERK-derived C1P is a general regulator of golgi fragmentation

To determine if CERK-derived C1P is a general regulator of Golgi fragmentation, human umbilical vascular endothelial cells (HUVECs) were chosen as the molecular tools are available and optimized to genetically modulate CERK and CPTP (50, 51, 54, 55, 59). HUVECs were treated with siRNAs to downregulate CPTP and CERK levels (SI Appendix, Fig. S2A). CPTP downregulation increased D-e-C_14:0_, D-e-C_16:0_, D-e-C_24:0_, and D-e-C_24:1_ C1P amounts versus non-targeting siRNA (siNT) treated cells (Fig. S2B). These results are like those observed for RF/6A cells (Fig. 3B). Conversely, CERK downregulation decreased levels of these C1P species (Fig. S2B), analogous to our findings with NVP231 in HL-60 and RF/6A cells (Fig. 2B; Fig. S1A). Additionally, siCERK and siCPTP co-knockdown (siCERK/siCPTP) did not significantly alter C1P levels (Fig. S2B), demonstrating that CPTP regulates CERK-derived C1P levels. To examine the effect of modulating C1P levels on Golgi morphology, CERK and CPTP were downregulated individually and simultaneously in HUVECs. Increasing C1P levels via CPTP downregulation induced fragmentation of both the *cis*- and *trans*-Golgi while reducing C1P via CERK downregulation promoted compact Golgi morphology (Fig. S2C through E). Simultaneous CERK and CPTP downregulation did not perturb Golgi morphology. Thus, CERK-derived C1P transported by CPTP regulates Golgi structure, which is a generalized mechanism for this bioactive lipid in multiple cell lines.

### Elevated C1P levels stimulate GRASP55 and GRASP65 phosphorylation

**Fig 4:**
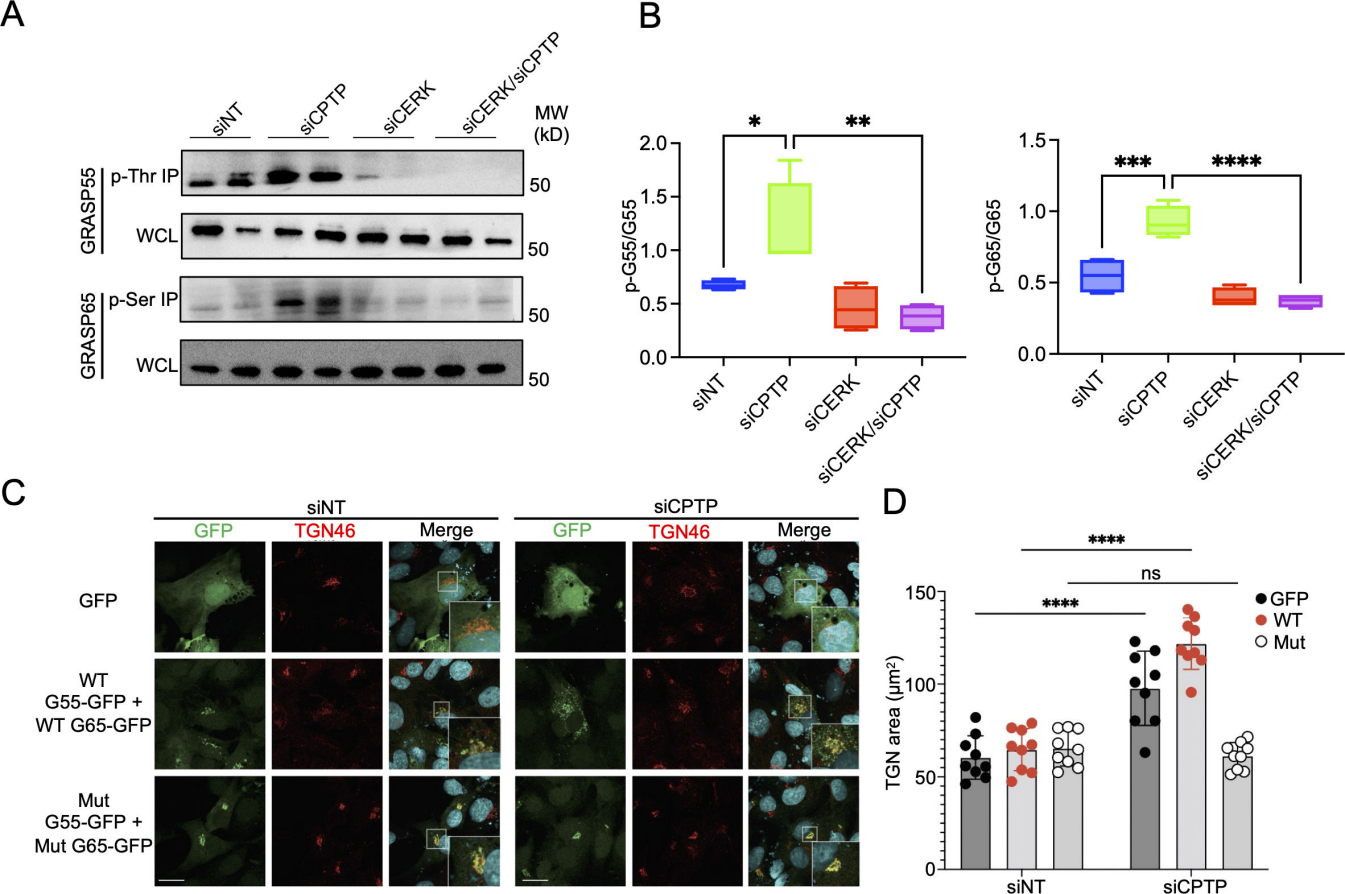
Induction of CERK-derived C1P stimulates GRASP55 and GRASP65 phosphorylation. (**A and B**) Elevating C1P levels promotes GRASP phosphorylation. HUVECs were transfected with siNT, siCPTP, siCERK, or both siCERK and siCPTP (siCERK/siCPTP). (**A**) To assess phosphorylation of GRASP55 and GRASP65, threonine-phosphorylated (p-Thr) and serine-phosphorylated (p-Ser) proteins immunoprecipitated (IP) from duplicate whole-cell lysates (WCLs) at 72 h post-transfection were Western-blotted and probed with antibodies specific for GRASP55 and GRASP65, respectively. WCLs were probed with GRASP55 and GRASP65 antibodies as loading controls. (**B**) Levels of phospho-GRASP55 (P-G55) and phospho-GRASP65 (P-G65) were assessed as the mean (±SD) normalized ratio of p-G55:GRASP55 (G55) and p-G65:GRASP65 (G65) densitometric signals. Data are representative of at least four independent experiments. Statistical analysis was performed using one-way ANOVA with Tukey’s post hoc test. (**C and D**) Golgi fragmentation is reduced in cells ectopically expressing phosphorylation-resistant GRASP55 and GRASP65. RF/6A cells were transfected to express WT GRASP55-GFP and WT GRASP65-GFP, phosphorylation-resistant GRASP55-GFP (TT225,249AA) and phosphorylation-resistant GRASP65-GFP (TSS216,220,274AAA) (Mut), or GFP. (**C**) The cells were fixed, immunolabeled with TGN46 antibody (Alexa Fluor 594 secondary, red), stained with DAPI to visualize host cell nuclei, and imaged using LSCM. Scale bar, 20 µm. (**D**) The mean (±SD) size of the Golgi area was calculated by measuring the Golgi in 25 GFP-expressing cells per coverslip in triplicate for three independent experiments. Data are for a total of 225 cells per condition. Statistical analysis was performed using two-way ANOVA and Sidak’s multiple comparison test. *P < 0.05; **P < 0.01; ***P < 0.001; and ****P < 0.0001.

Because phosphorylation of GRASP55 and GRASP65 promotes Golgi ribbon uncoupling (8–10, 19, 68, 80), we examined if C1P-induced Golgi fragmentation is linked to phosphorylation of these proteins. Serine- and threonine-phosphorylated proteins were immunoprecipitated from whole-cell lysates of CPTP and/or CERK siRNA-treated HUVECs and assessed for GRASP55 and GRASP55 by Western blot analysis. Increasing C1P levels by CPTP downregulation significantly induced phosphorylation of both proteins (Fig. 4A and B). Conversely, CERK downregulation nearly ablated this phenomenon and even did so in the presence of CPTP downregulation. To directly assess if C1P perturbs Golgi morphology via GRASP phosphorylation, HUVECs were transfected to co-express C-terminally GFP-tagged wild-type GRASP55 and GRASP65 or the alanine-substituted phosphorylation-resistant versions thereof, GRASP55-TT225,249AA and GRASP65-TSS216,220,277AAA (19, 81–86). Cells expressing GFP alone were a negative control. Treating each transfected population with siNT had no effect on TGN area while CPTP downregulation promoted marked increases in TGN area in cells expressing either GFP or both GRASP55-GFP and GRASP65-GFP (Fig. 4C). CPTP downregulation failed to alter TGN area in cells expressing GRASP55-TT225,249AA-GFP and GRASP65-TSS216,220,274AAA-GFP. Hence, CERK-derived C1P promotes Golgi structural changes by inducing GRASP55 and GRASP65 phosphorylation.

### C1P regulates Cdc42 membrane localization, and Cdc42 is critical for C1P to alter Golgi morphology and promote ApV maturation

**Fig 5:**
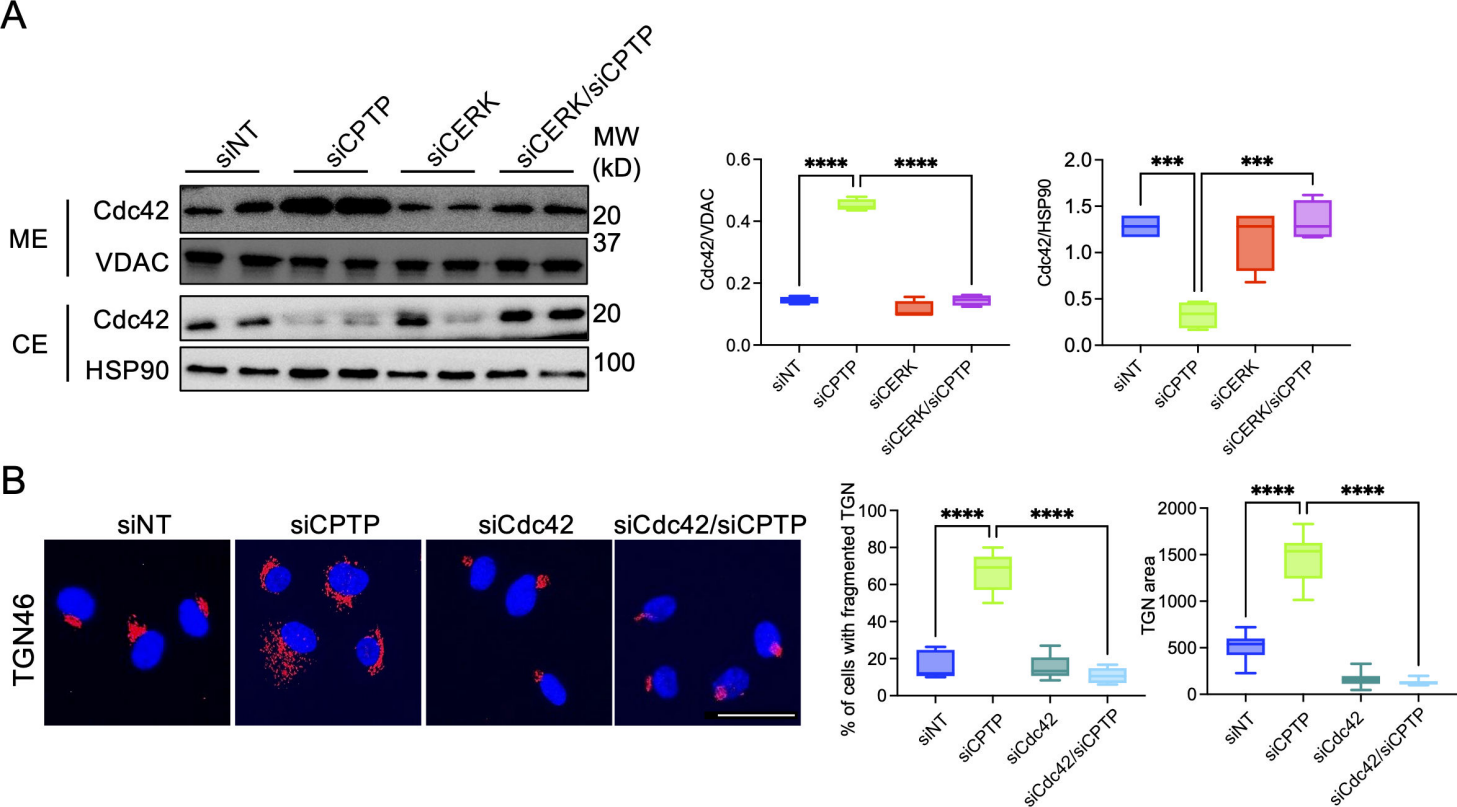
C1P induces Cdc42 membrane association, which is required for C1P-mediated Golgi morphological changes. (**A**) C1P induces Cdc42 membrane association. HUVECs were transfected with siNT, siCPTP, siCERK, or siCERK/siCPTP, followed by subcellular fractionation at 72 h. Cytosolic (CE) and membrane (ME) fractions were analyzed by Western blotting to determine the localization of Cdc42 as the mean (±SD) normalized ratio of Cdc42:voltage-dependent anion channel (VDAC, ME associated protein) and Cdc42: heat shock protein 90 (HSP90, CE associated protein) densitometric signals. Data are representative of four independent experiments. Statistical analysis was performed using one-way ANOVA, followed by Tukey’s post hoc test. (**B**) Cdc42 downregulation prevents C1P-induced Golgi fragmentation. HUVECs were transfected with siRNAs as (**A**). At 72 h, the cells were fixed, immunolabeled with TGN46 antibody (Alexa Fluor 594 secondary, red), stained with DAPI (blue) to label host cell nuclei, and imaged with immunofluorescence microscopy. Scale bar, 20 µm. Fluorescence micrographs were examined to determine the mean (±SD) percentage of cells with dispersed Golgi and mean (±SD) Golgi area. Data are representative of four independent experiments in which 50 cells were examined per condition each time. Statistical analysis was performed using a one-way ANOVA with Tukey’s post hoc test. ***P < 0.001 and ****P < 0.0001.

Cdc42 is a key regulator of COPI vesicle trafficking as well as Golgi morphology (87). Disrupting generation of the C1P precursor molecule, ceramide, impedes Cdc42 membrane localization, and activity (88, 89). When coupled with our findings that C1P produces the opposite effect of ceramide on Golgi morphology, and Cdc42 regulates processes that are attributed to C1P—Golgi fragmentation, GRASP55 and GRASP65 phosphorylation, and COPI vesicle trafficking (11, 13, 14, 90–93)—we analyzed the effect of modulating C1P levels on Cdc42 membrane localization. Elevating C1P levels by CPTP downregulation in HUVECs significantly induced Cdc42 membrane localization, whereas C1P downregulation via siCERK or siCERK/siCPTP treatment decreased Cdc42 membrane localization (Fig. 5A). siRNA-mediated downregulation of Cdc42 (Fig. S3) blocked the ability of increased C1P levels to alter Golgi morphology (Fig. 5B). Thus, CERK-derived C1P promotes Cdc42 recruitment to cellular membranes. Furthermore, the induction of Golgi fragmentation by CERK-derived C1P requires Cdc42.

**Fig 6:**
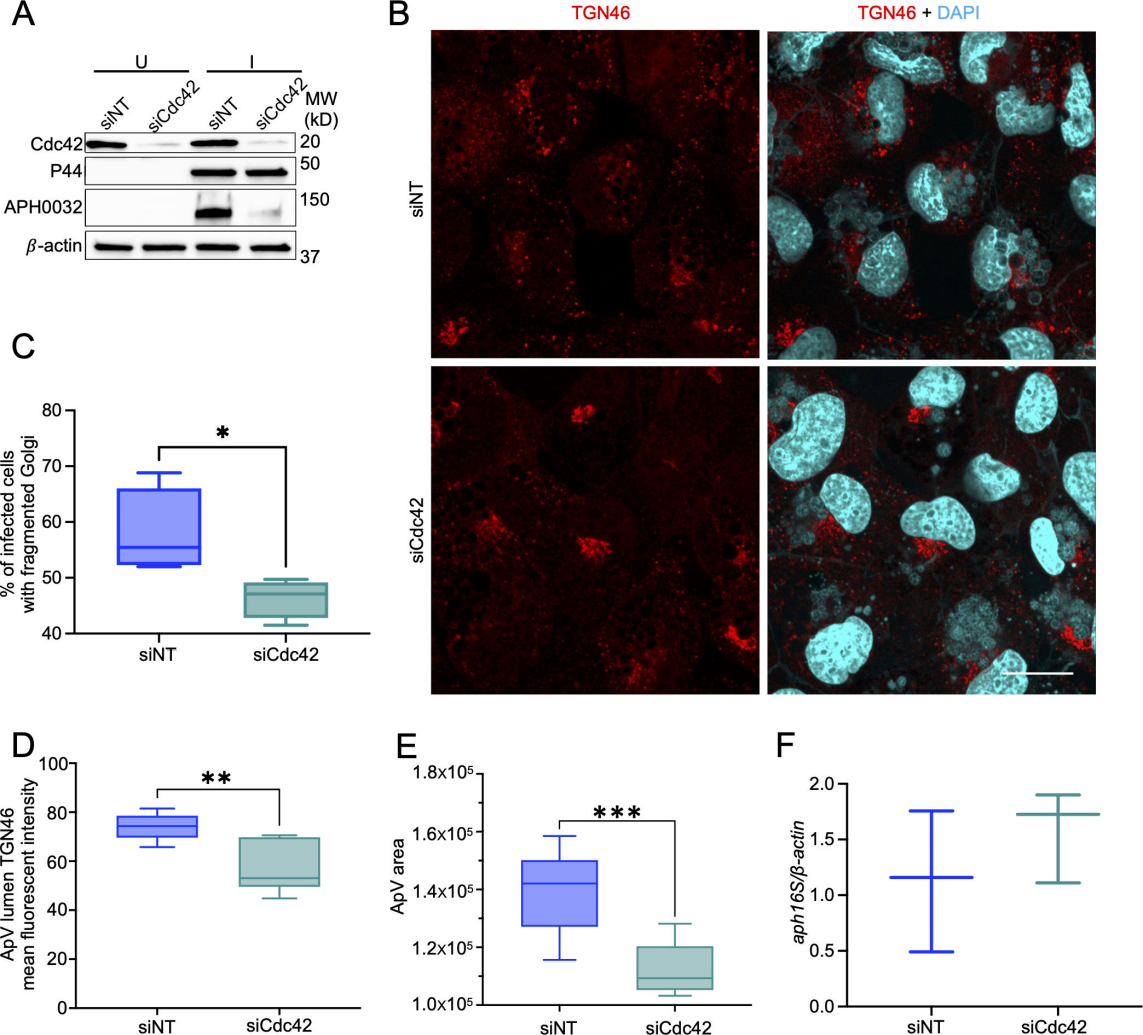
Cdc42 downregulation inhibits delivery of *trans*-Golgi derived vesicles into the ApV and impairs ApV maturation. RF/6A cells were transfected with siNT or siCdc42. At 48 h, the cells were either left uninfected or infected with *A. phagocytophilum* DC organisms. At 24 h post-infection, whole-cell lysates were subjected to Western blotting with the antibodies specific for Cdc42, *A. phagocytophilum* outer membrane prorein P44, *A. phagocytophilum* effector protein APH0032, or β-actin (**A**). Data are representative of at four separate experiments. (B–E) Duplicate samples were fixed, immunolabeled with TGN46 antibody (Alexa Fluor 594 secondary, red), stained with DAPI (blue) to visualize host cell nuclei and bacterial nucleoids, and imaged using LSCM (***B***). Scale bar, 20 µm. Immunofluorescence micrographs were examined to determine the mean (±SD) percentage of infected cells with fragmented Golgi (**C**), TGN46 immunosignal mean (±SD) fluorescent intensity inside ApV lumen (**D**), and mean (±SD) ApV area (**E**). Fifty cells were examined in triplicate per condition. Data are representative of at least three separate experiments. (**F**) DNA isolated from siNT- or siCdc42-treated RF/6A cells that had been infected with *A. phagocytophilum* or not was subjected to qPCR to determine the bacterial load as the normalized *aph16s:β-actin* ratio using the 2^−ΔΔCT^ method. Data are representative of four separate experiments. Statistical analyses were performed using an unpaired, two-tailed *t* test with Welch’s correction. *P < 0.05; **P < 0.01; and ***P < 0.001.

To examine if Cdc42-regulated Golgi disruption benefits *A. phagocytophilum* infection, siCdc42 or siNT-treated RF/6A cells (Fig. 6A) were incubated with DC organisms. At 24 hpi, Golgi fragmentation was inhibited by 16% and TGN immunosignal in the ApV lumen was reduced by 22% in Cdc42 knockdown cells (Fig. 6B through D). Concomitantly, Cdc42 downregulation had no effect on the bacterial load, but significantly, albeit modestly, impaired ApV expansion (Fig. 6E and F). Also, Cdc42 downregulation nearly abrogated *A. phagocytophilum* expression of APH0032 (Fig. 6A), a secreted effector protein that is upregulated and localizes to the ApV membrane during ApV maturation prior to DC release (25, 71, 72). Thus, while Cdc42 downregulation does not inhibit *A. phagocytophilum* replication, it reduces TGN anterograde traffic into the ApV, which is linked to an inhibition in ApV maturation and hence progression of the pathogen’s infection cycle.

### C1P modulates the PKCα/Cdc42/JNK signaling axis

**Fig 7:**
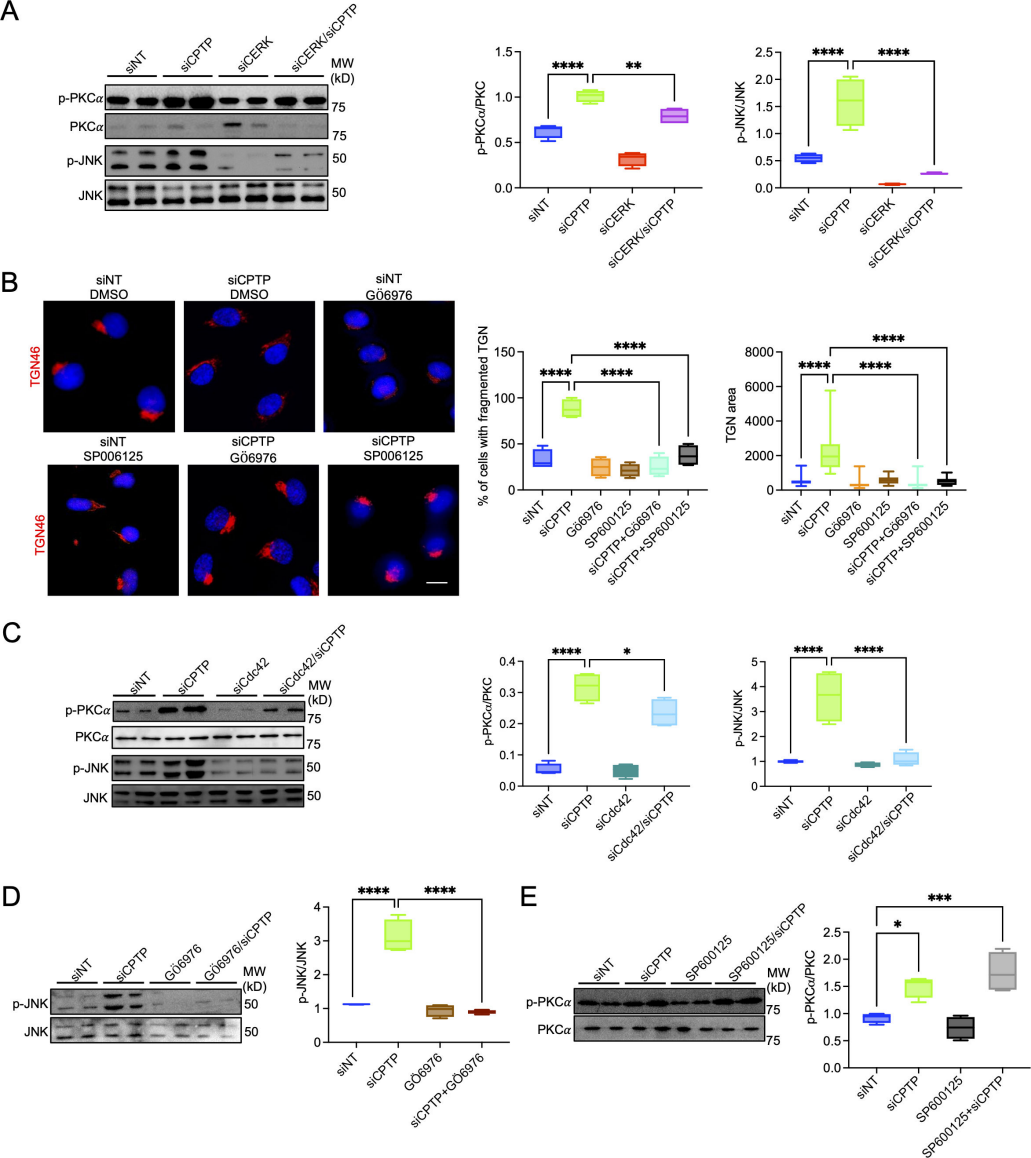
PKCα and JNK are required for C1P-stimulated changes in Golgi morphology. (**A**) C1P induces PKCα and JNK phosphorylation. HUVECs were transfected with siNT, siCPTP, siCERK, or siCERK/siCPTP. At 72 h, phosphorylated PKCα (p-PKCα), p-JNK, total PKCα, and total JNK levels were analyzed by Western blotting. Levels of p-PKCα and p-JNK were assessed as the mean (±SD) normalized ratios of p-PKCα:PKCα and p-JNK:JNK densitometric signals. (**B**) PKCα and JNK inhibition blocks C1P-induced Golgi dispersal. HUVECs transfected with siNT or siCPTP cells were treated at 48 h post-siRNA addition with 3 µM Gö6976 or 25 µM SP006125. At 24 h post-treatment, the cells were fixed, immunolabeled with antibody against TGN46 (Alexa Fluor 594 secondary, red), stained with DAPI (blue) to visualize host cell nuclei, and examined by LSCM. Scale bar, 20 µm. Fluorescence micrographs were examined to determine the mean (±SD) percentage of cells with fragmented Golgi and mean (±SD) Golgi area. Data presented are representative of four independent experiments in which 50 cells were examined per condition each time. (**C**) Cdc42 downregulation inhibits C1P-induced JNK phosphorylation, but not PKCα. HUVECs were transfected with siNT, siCPTP, siCdc42 or siCdc42/CPTP. At 72 h, p-PKCα, p-JNK, total PKCα, and total JNK levels were analyzed by Western blotting and densitometry to determine the mean (±SD) normalized ratios of p-PKCα:PKCα and p-JNK:JNK. (**D and E**). PKCα is upstream of JNK in C1P-mediated signaling. HUVECs that had been treated with siNT or siCPTP were treated with Gö6976 (**D**) or SP006125 (**E**). At 24 h post-treatment, the mean (±SD) normalized ratios of P-PKCα:PKCα and p-JNK:JNK were determined. Data are representative of four independent experiments. Statistical analyses were performed using one-way ANOVA with Tukey’s post hoc test. *P < 0.05; **P < 0.01; ***P < 0.001; and ****P < 0.0001.

PKCα is an upstream regulator of JNK and promotes its recruitment to cellular membranes (94–102). Cdc42 is also as an upstream activator of JNK (11, 103–106). PKCα and JNK regulate phosphorylation of GRASP55 and GRASP65, respectively (19, 68). Furthermore, the addition of exogenous C1P to cells activates JNK and PKCα signaling (62, 69). Due to these signaling links, we examined the role of CERK-derived C1P in activating JNK and PKCα as well as inducing phosphorylation of GRASP55 and GRASP65. Elevating C1P levels in HUVECs by CPTP downregulation elicited both PKCα and JNK phosphorylation, and both of which were significantly inhibited by CERK downregulation (Fig. 7A). Inhibition of PKCα using Gö6976 and JNK using SP600125 each blocked the ability of CERK-derived C1P to disrupt Golgi morphology (Fig. 7B).

To orient the signaling cascade involving Cdc42, PKCα, and JNK that results in C1P-induced Golgi fragmentation, we downregulated Cdc42 and assessed its effect on C1P-induced PKCα and JNK phosphorylation. Depleting Cdc42 modestly impaired C1P-induced PKCα phosphorylation but did not affect the total induction of PKCα phosphorylation when accounting for the suppression of basal PKCα phosphorylation by Cdc42 downregulation alone (Fig. 7C). On the other hand, the induction of JNK phosphorylation by C1P was abolished by Cdc42 downregulation suggesting that Cdc42 is downstream of PKCα and upstream of JNK. As PKCα also activates JNK (94–102), we investigated if C1P induces JNK phosphorylation by PKCα-dependent signaling. Gö6976 pronouncedly repressed C1P-mediated JNK phosphorylation (Fig. 7D). In contrast, SP600125 had no effect on C1P-mediated PKCα phosphorylation (Fig. 7E). These data show that C1P stimulates PKCα activation followed by activation of Cdc42 and then JNK.

**FIG 8:**
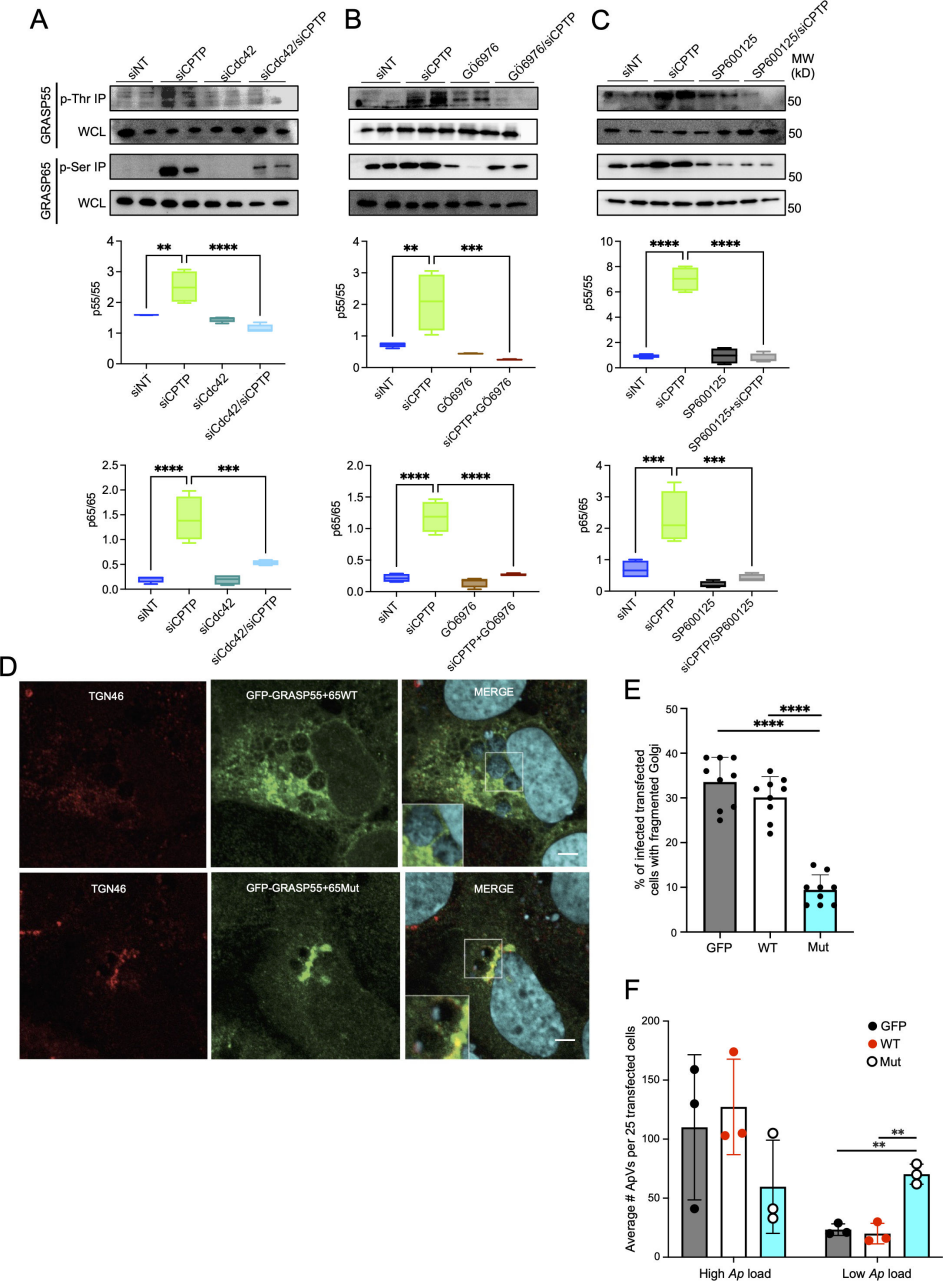
C1P induces Golgi fragmentation via Cdc42/PKCα/JNK regulated phosphorylation of GRASP55 and GRASP65 that is critical for *A. phagocytophilum* infection. (A–C). Cdc42, PKC, and JNK each promote GRASP phosphorylation. HUVECs were transfected with siNT, siCPTP, siCdc42, or siCdc42/CPTP (**A**). At 48 h, siCPTP-treated cells were incubated with control (0.001% DMSO), 3 μM Gö6976 (**B**), or 25 µM SP006125 (**C**). At 24 h post-treatment, threonine-phosphorylated (p-Thr) and serine-phosphorylated (p-Ser) proteins immunoprecipitated (IP) from duplicate whole-cell lysates (WCLs) were Western-blotted and probed with antibodies specific for GRASP55 and GRASP65, respectively. WCLs were probed with GRASP55 and GRASP65 antibodies as loading controls. Levels of phospho-GRASP55 (P-G55) and phospho-GRASP65 (P-G65) were assessed as the mean (±SD) normalized ratio of p-G55:GRASP55 (G55) and p-G65:GRASP65 (G65) densitometric signals. Data are representative of four independent experiments. Statistical analysis was performed using one-way ANOVA with Tukey’s post hoc test. (D–F) GRASP phosphorylation is critical for *A. phagocytophilum*-induced Golgi fragmentation and optimal *A. phagocytophilum* fitness. RF/6A cells expressing WT GRASP55-GFP and WT GRASP65-GFP, phosphorylation-resistant GRASP55-GFP (TT225,249AA) and phosphorylation-resistant GRASP65-GFP (TSS216,220,274AAA), or GFP were infected with *A. phagocytophilum* DC organisms. (**D**) At 24 h, the cells were fixed, immunolabeled with TGN46 antibody (Alexa Fluor 594 secondary, red), stained with DAPI (blue) to denote host cell nuclei and bacterial nucleoids, and examined by LSCM. Scale bar, 10 µm. (**E**) The mean (±SD) percent of infected GFP-positive cells with fragmented Golgi was determined by examining 150 cells total per condition for three independent experiments. Data are the combined total of 450 cells examined per condition from the three experiments. (**F**) The mean (±SD) number of ApVs exhibiting high (full of bacteria) or low (one to two bacteria) bacterial loads per 25 GFP-positive cells was determined. Data are for three independent experiments. Each black, red, or white circle corresponds to the mean number of ApVs in GFP-expressing cells per experiment. Statistical analyses for panels (E and D) were performed using one-way ANOVA with Tukey’s post hoc test. **P < 0.01; ***P < 0.001; and ****P < 0.0001.

To examine if the C1P-mediated PKCα/Cdc42/JNK pathway induces GRASP55 and GRASP65 phosphorylation, HUVECs were treated with siCdc42, Gö6976, or SP600125 independently or in conjunction with siCPTP. Threonine- and serine-phosphorylated proteins recovered by immunoprecipitation were subjected to Western blot analysis to assess total and phosphorylated forms of GRASP55 and GRASP65. Cdc42, PKCα, and JNK inhibition each markedly reduced C1P-induced phosphorylation of GRASP55 and GRASP65 (Fig. 8A through C). Taken together, these results show that C1P induces the PKCα/Cdc42/JNK signaling pathway that ultimately results in GRASP phosphorylation and subsequent disruption of the Golgi apparatus.

### *A. phagocytophilum* requires GRASP55 and GRASP65 phosphorylation to promote Golgi fragmentation and for optimal infection

Because C1P stimulates signaling that culminates in GRASP phosphorylation and Golgi destabilization, and since *A. phagocytophilum* promotes Golgi fragmentation as a promicrobial strategy (25), we evaluated if the bacterium induces Golgi structural changes in a GRASP phosphorylation-dependent manner. RF/6A cells were transfected to express GRASP55-GFP and GRASP65-GFP, phosphorylation-resistant mutants thereof, or GFP alone, followed by incubation with DC organisms. At 24 h, the percentage of *A. phagocytophilum* infected cells co-expressing GRASP55-TT225,249AA-GFP and GRASP65-TSS216,220,277AAA-GFP that had fragmented Golgi was reduced by at least 70% versus controls (Fig. 8D and E). Moreover, ApVs in cells expressing phosphorylation-resistant GRASP55 and GRASP65 harbored markedly fewer bacteria than in cells co-expressing GFP-tagged wild-type GRASP55 and GRASP65 or GFP (Fig. 8D and F). Therefore, *A. phagocytophilum* perturbs Golgi morphology in a GRASP phosphorylation-dependent manner, which, in turn, is critical for optimal proliferation within its vacuolar niche.

## DISCUSSION

**Fig 9:**
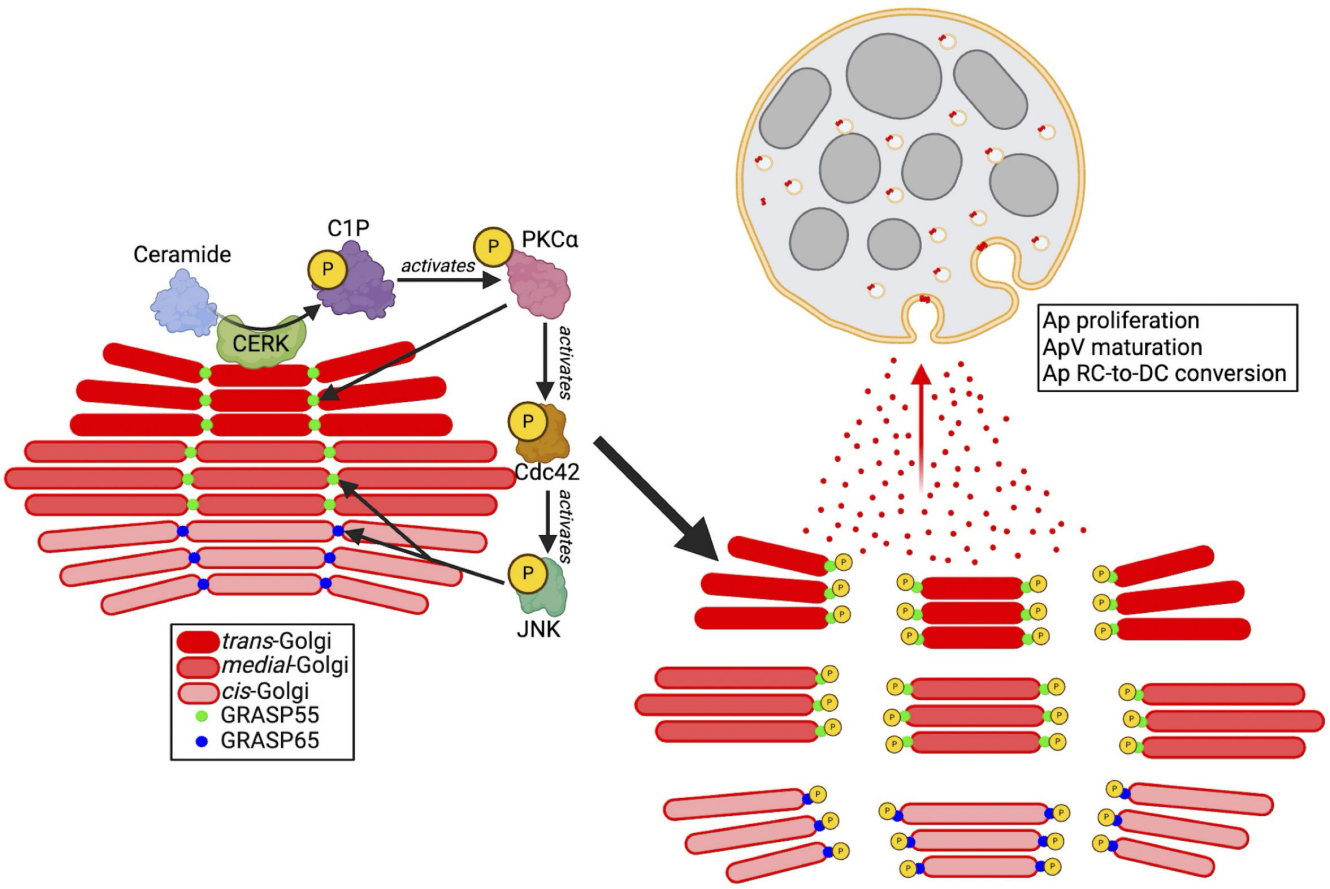
Model. *A. phagocytophilum* elevates host cell C1P levels, which induces signaling through PKCα, Cdc42, and JNK that leads to GRASP55 and GRASP65 phosphorylation that, in turn, promotes uncoupling of the Golgi ribbon. The resulting increase in anterograde trafficking of TGN-derived vesicles into the ApV, a pathogen-modified MVB, benefits the *A. phagocytophilum* infection cycle.

Obligate intracellular pathogens are master cell biologists that exploit host processes to their advantage and can therefore serve as useful tools for elucidating unrecognized eukaryotic cellular pathways. By studying *A. phagocytophilum*, we revealed a role for CERK-derived C1P as a regulator of Golgi structure. C1P induces signaling through PKCα, Cdc42, and JNK that leads to GRASP phosphorylation and, consequently, perturbation of Golgi morphology (Fig. 8). When these findings are considered together with prior reports that (i) fragmented Golgi exhibit accelerated anterograde trafficking (1), (ii) GRASP phosphorylation or depletion increases Golgi anterograde trafficking (7, 107), (iii) MVBs receive anterograde traffic from the TGN (45), and (iv) *A. phagocytophilum* not only lives in a pathogen-modified MVB but also parasitizes TGN-derived vesicles that are delivered into the ApV lumen (25, 44), the essentiality of C1P to the bacterium’s infection cycle becomes clear. By elevating host cell C1P levels and coopting the C1P-induced signaling axis, *A. phagocytophilum* upregulates TGN vesicle delivery into its parasitophorous vacuole, which increases the bacterial load, promotes ApV maturation, and is key for RC-to-DC conversion (Fig. 9). Pharmacologic inhibition and siRNA-mediated downregulation of CERK pronouncedly lower *A. phagocytophilum* levels and bacterial expression of the DC marker, *aph1235*. Notably, treatment with siRNA targeting Rab10, which mediates anterograde trafficking from the TGN, has the same effect (25). The importance of C1P to *A. phagocytophilum* is recapitulated *in vivo*, as it fails to infect CERK^−/−^ mice. Conversely, raising C1P levels via CPTP downregulation increases bacterial levels and ApV numbers per cell. Because each ApV derives from the entry of a single *A. phagocytophilum* organism (43), the increase in ApVs per cell could be due to the acceleration of the infection cycle leading to more reinfection events. Alternatively, it could result from a Golgi fragmentation-associated increase in the trafficking of receptors critical for *A. phagocytophilum* entry to the cell surface. How *A. phagocytophilum* elevates C1P levels is unclear, but potential unexplored or understudied mechanisms include activating CERK, decreasing C1P transport, or suppressing C1P catabolism.

D-e-C14:0 C1P and D-e-C16:0 C1P levels are elevated in both *A. phagocytophilum* infected cells and cells in which CPTP has been depleted using siRNA. There have been no reports for specific chain lengths of C1P having variable functions. Any differential acyl chain lengths for C1P are currently surmised to follow the available substrate ceramide for CERK and could be indicative of cellular location (54, 56, 57, 108), but this has not been strongly interrogated. To date, studies have been hampered by the low levels of C1P in many cell types allowing for the main forms of C1P, which are D-e-C14:0 C1P and D-e-C16:0 C1P, to be appropriately quantitated (50, 51, 54, 109).

CERK-derived C1P activates Cdc42 to enhance its translocation from the cytoplasm to cellular membranes. Downregulation of Cdc42 abrogates the effect of C1P on the induction of Golgi destabilization showing that Cdc42 is a key factor in the downstream signaling that C1P modulates. Moreover, Cdc42 downregulation also inhibits ApV maturation as shown by a lack of APH0032 induction and reduction in ApV area. A functional role of APH0032 has not been discerned. Whether Cdc42 downregulation impairs the expression of APH0032 exclusively or other unidentified AVM-localized *A. phagocytophilum* effectors that are coincidentally expressed with APH0032 during the infection cycle is unknown. If the latter is true, then Cdc42 could play a significant role in influencing ApV maturation and *A. phagocytophilum* pathogenesis. Additionally, Cdc42 regulation of actin-related processes, which are targeted by numerous intracellular pathogens (110–112), cannot be ruled out as contributing to *A. phagocytophilum* intracellular fitness. Why Cdc42 downregulation modestly inhibits Golgi fragmentation in RF/6A cells, but pronouncedly does so in HUVECs and why inhibiting or downregulating CERK strongly impairs this mechanism in both cell lines is unclear. Specifically, our findings suggest that either Golgi fragmentation regulates only the ApV maturation process of the *A. phagocytophilum* infection cycle or C1P can facilitate Golgi fragmentation in RF/6A cells by additional pathways independent of Cdc42. PKCα is known to directly phosphorylate GRASP55 (19), which would induce Golgi ribbon uncoupling. This alternate/cooperating mechanism of GRASP phosphorylation would be Cdc42-independent and explain the significant, but minimal effect of Cdc42 downregulation on *A. phagocytophilum*-induced Golgi fragmentation in RF/6A cells. Indeed, activation of PKCα at the Golgi apparatus could bypass the need for Cdc42 activation to induce Golgi instability and directly induce GRASP55 phosphorylation. This report supports both plausible mechanisms. Moreover, it suggests a cooperative role between PKCα direct phosphorylation of GRASP55 and the indirect signaling cascade of PKCα activation of Cdc42 and subsequent GRASP phosphorylation (Fig. 8).

Prior to this report, whether *A. phagocytophilum* infection activates JNK signaling was unknown. Herein, we oriented the signal transduction pathway from PKCα to Cdc42 to JNK downstream of the pathogen inducing an increase in CERK-derived C1P. We further showed that a key biological mechanism modulated by JNK that drives the *A. phagocytophilum* infection cycle is GRASP65 phosphorylation and ensuing Golgi fragmentation. JNK^−/−^ mice are highly resistant to *A. phagocytophilum* infection, and this has been linked to the repressive role of JNK on CD1d-restricted natural killer T cells production of IFNγ, a cytokine that is critical for clearing the infection in mice (113). In lieu of our findings, it is expected that *cis*-Golgi uncoupling induced by the JNK-GRASP65 axis would be inhibited in JNK^−/−^ mice, which would at least partially impair the pathogen’s ability to parasitize anterograde traffic and synergize with IFNγ-mediated clearing.

How CERK-derived C1P activates PKCα to facilitate subsequent activation of Cdc42 and JNK is not known, but PKCα possesses a C2-domain (114, 115). C1P binds and activates group IVA phospholipase A_2_ via this enzyme’s C2 domain facilitating its translocation to the Golgi (49–52, 57, 58, 61, 116, 117). This gives rise to the hypothesis that C1P analogously binds PKCα at its C2-domain to translocate PKCα to the Golgi. The possibility that C1P associates with PKCα may explain the conundrum as to how classical PKCs translocate to different membranes in response to specific agonists. For example, classical PKCs prefer the phosphatidylserine (PS)-rich plasma membrane when acutely activated versus internal membranes. Alternatively, sustained activation of classical PKCs such as PKCα induces the translocation of these enzymes to a pericentriolar region/centrioles, a site of mitotic signaling, and Golgi fragmentation is a key mechanism in mitosis (118–125). The link between C1P, PKCα, and Golgi fragmentation established herein may explain the known role of C1P in cellular proliferation (46, 55, 69).

Inducing changes in Golgi morphology is a broadly conserved strategy among diverse pathogens. Yet, the underlying mechanisms are not well understood and only a few targeted host cell factors are known (22–40). Many of those that have been identified are Golgi matrix and tethering proteins. *Chlamydia trachomatis* promotes cleavage of golgin-84 (31). *Legionella pneumophila* uses its effectors LegA15 to dislocate p115 from the Golgi and SdeA to phosphoribosyl-ubiquitinate GRASP55 and GRASP65 to prevent their oligomerization (23, 38). Whereas *Chlamydia* and *Legionella* directly promote Golgi disassembly by targeting proteins responsible for maintaining the organelle’s integrity, *A. phagocytophilum* indirectly does so through CERK-derived C1P. Human cytomegalovirus induces GRASP65 phosphorylation by an unknown means to fragment the Golgi as an essential step in infectious particle production (30). SARS-CoV-2 triggers Golgi dispersion by downregulating GRASP55 to accelerate viral trafficking and release (29, 36, 126). Interestingly, interaction of the SARS-CoV-2 spike protein with host angiotensin-converting enzyme 2 that mediates invasion is enhanced by heparan sulfate, the synthesis of which is increased by GRASP depletion (127–129). Furthermore, a major cause of Golgi morphological changes in Alzheimer’s disease is GRASP65 phosphorylation (107). Given that SARS-CoV-2 can cause a neuropathological phenotype reminiscent of Alzheimer’s Disease as well as clinical brain fog, it has been speculated that SARS-CoV-2 neuropathology might be caused by viral-induced GRASP phosphorylation (127). As the host factors that drive Golgi fragmentation associated with the progression of other neurodegenerative diseases, inflammatory disorders, and cancers are incompletely defined (1, 20), the potential contribution of C1P to these conditions is worth considering.

Our study provides the foundation to explore C1P as an antimicrobial target to treat HGA as well as other infectious and non-infectious diseases whose pathologies are associated with GRASP-dependent Golgi structural changes. Importantly, CERK inhibitors are well tolerated in mice (50). As precedent for host-directed therapeutics against *A. phagocytophilum*, tricyclic antidepressants that inhibit aSMase, a key enzyme for the bacterium’s intra-MVB/TGN parasitic lifestyle, halt *A. phagocytophilum* infection (66, 67). Future studies are needed to determine how C1P activates both Cdc42 and PKCα along with the mechanism by which *A. phagocytophilum* elevates CERK-derived C1P levels as little is known regarding CERK activation and sustained C1P levels in specific cellular topologies. In closing, our work reveals a previously unappreciated role for C1P in regulating Golgi dispersal, a cellular pathology linked to the progression of diverse diseases and identifies the bioactive sphingolipid as a host factor that is critical for *A. phagocytophilum* pathogenesis.

## MATERIALS AND METHODS

### Cell culture and drug treatments

Uninfected and *A. phagocytophilum* (NCH-1 strain) infected human promyelocytic HL-60 cells [ATCC, CCL-240; American Type Culture Collection (ATCC)] and *Macaca mulatta* RF/6A choroidal endothelial cells (CRL-1780; ATCC) were cultured as described in (71). HL-60 and RF/6A cells pretreated with 300 nM and 400 nM of NVP231 [Cayman Chemical, (catalog #13858)], respectively, 50 µM of SP006125 [Sigma Millipore (372770)], 3 µM of Gö6976 (Sigma Aldrich, 372770), or 0.001% of DMSO for 1 h were incubated with *A. phagocytophilum* as described in reference (70). HUVECs (Lonza, CC-2519) were cultivated in Endothelial Cell Growth Medium-2 Bullet Kit (Lonza) in a humidified incubator at 37°C with 5% atmospheric CO_2_.

### siRNA and plasmid transfection

Cdc42, CERK, and CPTP were knocked down using two sequence-specific siRNAs (Dharmacon) as described in references (49, 51). The siRNAs were transfected into HUVECs using Dharmafect 4 transfection reagent (Dharmacon) per the manufacturer’s instruction. The cells were harvested after 48 h for RNA extraction and after 72 h for Western immunoblotting and lipidomic analyses. pEGFP-VSVGtsO45 was a gift from Dr. Jennifer Lippincott-Schwartz (Addgene plasmid #11912; http://n2t.net/addgene:11912; RRID:Addgene_11912). pEGFP-N1-GRASP55 (Addgene plasmid #137708; http://n2t.net/addgene:137708; RRID:Addgene_137708), and pEGFP-N2-GRASP65 (Addgene plasmid #137709; http://n2t.net/addgene:137709; RRID:Addgene_137709) were gifts from Dr. Yanzhuang Wang. Lipofectamine 3000 (Invitrogen) and Lipofectamine LTX (Invitrogen) were used to transfect RF/6A cells and HUVECs, respectively.

### RNA extraction and RT-qPCR analyses

RNA was extracted using the RNeasy minikit (Qiagen) and reverse-transcribed into cDNA using the Applied Biosystems High-Capacity cDNA Reverse Transcription kit (ThermoFisher). Using cDNA as template, qPCR was performed with Taqman Universal PCR Master Mix (ThermoFisher) and primers specific for *CERK* (ThermoFisher Hs00368483_m1), *CPTP* (ThermoFisher Hs00257998_s1), *β-actin* (72), *A. phagocytophilum 16S rDNA* (72), and *A. phagocytophilum aph1235* (73). Thermal cycling conditions were as previously described (54). Relative expression among samples was determined using the 2^−ΔΔCT^ method (130) in which CERK, CPTP, and *A. phagocytophilum 16S rDNA* expression were normalized to that of *β-actin* and *aph1235* expression was normalized to that of *A. phagocytophilum 16S rDNA*.

### Western immunoblotting

Western immunoblotting was performed as previously described (49). Primary antibodies used to detect proteins of interest were anti-VDAC [Cell Signaling Technology (RRID:AB_10557420); 1:1,000], anti-β-actin [Cell Signaling Technology (RRID:AB_2242334); 1:1,000], anti-Cdc42 [Cell Signaling Technology (RRID:AB_2078085); 1:500], anti-phospho-JNK [Cell Signaling Technology (RRID:AB_823588); 1:500], anti-JNK [Cell Signaling Technology (RRID:AB_2250373); 1:1,000], anti-HSP90 [Cell Signaling Technology (RRID:AB_2233307); 1:2,000], anti-GRASP55 [Santa Cruz (RRID:AB_10708723); 1:500], anti-GRASP65 [Novus Biological (RRID:AB_2916091); 1:500], anti-phospho-threonine [Cell Signaling Technology (9381); 1:500], and anti-phospho-serine [Cell Signaling Technology (9631s); 1:500]. Subcellular fractionation was performed using Thermo Scientific Subcellular Protein Fractionation Kit for Cultured Cells (ThermoFisher) following the manufacturer’s instructions. Densitometric values were quantified using Fiji ImageJ (131) or a Chemidoc Touch Imaging System (Bio-Rad) and Image Lab 6.0 software (Bio-Rad).

### Immunofluorescence microscopy

Cells were fixed with 4% (vol/vol) paraformaldehyde (Electron Microscopy Sciences) in phosphate-buffered saline (PBS) for 20 min, followed by washing three times with ice-cold PBS. The cells were permeabilized with 0.25% (vol/vol) Triton X-100 (Fisher Scientific) in PBS and washed three times with ice-cold PBS. The cells were blocked with 10% (vol/vol) goat serum in PBS for 30 min or 5% (vol/vol) BSA in PBS for 30 min, followed by probing with primary antibodies diluted in 1% (vol/vol) goat serum or 1% (vol/vol) BSA for 90 min. Primary antibodies were rabbit anti-TGN46 [Novus Biologicals (RRID:AB_10011762); 1:500], Calreticulin [Thermo Fisher Scientific (PA3-16862); 1:200], GFP [Thermo Fisher Scientific (A-21311); 1:500], and GM130 [Novus Biological (RRID:AB_2916095); 1:200]. After washing with PBS, samples were incubated with secondary antibodies conjugated to Alexa Fluor fluorochromes (Invitrogen) in 1% (vol/vol) BSA for 1 h. DAPI (4′,6′-diamidino-2-phenylindole, Vesterfield) was used per the manufacturer’s instructions to stain host nuclei and *A. phagocytophilum* nucleoids. Coverslips were mounted using Prolong Gold Anti-fade reagent (Invitrogen) and imaged at room temperature. Samples were imaged using a Keyence BZX-800 microscope; a TCS SP8 microscope (Leica Microsystems) affixed with an Andor iXon Life 888 EMCCD camera (Oxford Instruments) and a 63× water-immersion objective with 1.2 numeric aperture; or a Zeiss LSM laser scanning confocal microscope (Zeiss). For quantifying increases in Golgi area in fluorescence micrographs obtained using the Keyence BZX-800 microscope, ImageJ was used to measure Golgi area in pixels from 50 cells per condition. For quantifying increases in Golgi area in fluorescence micrographs obtained using the TCS SP8 microscope, LAS X (Leica Microsystems) software (version 3.7.4.23463) was used to measure Golgi area in μm^2^ from 25 cells per condition in triplicate. ApV lumen TGN46 fluorescence intensity was determined using ImageJ. The *A. phagocytophilum* load in ApVs per 25 GFP-positive cells per condition and ApV area were assessed by examining fluorescence micrographs obtained using the TCS SP8 microscope using LAS X software.

### Sphingolipid analyses

Total cell lipids were harvested, sphingolipids extracted, and UPLC-ESI-MS/MS analyses were performed to quantitate sphingolipids as described (49–52, 54, 55, 109).

### Mouse studies

All mouse studies were undertaken under the supervision and approval of the USF IACUC (Protocol# IS00004094), an accredited AAALAC program (#000434). Male and female CERK^+/+^ and CERK^−/−^ littermate mice on a C57Bl/6 genetic background (50) (7–8 weeks of age) were injected intraperitoneally with 1 × 10^8^ *A. phagocyopilum* DC organisms as described (67). Blood was collected from the saphenous vein on days 4, 8, 12, 16, 21, and 24 post-injection, followed by the addition of heparin (Sigma-Aldrich) at 100 U mL^−1^. The peripheral blood *A. phagocytophilum* load was analyzed by qPCR as previously described (132). Mice were euthanized on day 28.

### Statistical analysis

Statistical analyses were performed using the Prism 8.0 software package (GraphPad, San Diego, CA). Statistical significance was set at *P* values of <0.05.

## Data Availability

All mass spectrometry lipidomics data have been deposited to NIH Common Fund’s National Metabolomics Data Repository (NMDR) website, the Metabolomics Workbench, https://www.metabolomicsworkbench.org. The Study ID is pending. The data can be accessed directly through the pending Project DOI and this NMDR repository is supported by NIH grant U2C-DK119886. All other data needed to evaluate the conclusions in the paper are present in the paper or the Supplementary Materials. The mouse models are available from the Chalfant Laboratory at the University of Virginia through a material transfer agreement Virginia Commonwealth University.

## AFTER EPUB

*[This article was published on 28 February 2024 with errors in Fig. 1. The figure was corrected in the current version, posted on 29 March 2024.]*

## SUPPLEMENTAL MATERIAL

Figure S1 mbio.00299-24-s0001.tiff CERK-derived C1P is critical for *A. phagocytophilum*-induced Golgi fragmentation and optimal infection in myeloid host cells.

Figure S2 mbio.00299-24-s0002.tiff CERK-derived C1P is a general regulator of Golgi morphology.

Figure S3 mbio.00299-24-s0003.tiff Confirmation of siRNA mediated Cdc42 downregulation.

ASM does not own the copyrights to Supplemental Material that may be linked to, or accessed through, an article. The authors have granted ASM a non-exclusive, world-wide license to publish the Supplemental Material files. Please contact the corresponding author directly for reuse.
