# Supplementary figures and images for: Ceramide-1-phosphate is a regulator of Golgi structure and is co-opted by the obligate intracellular bacterial pathogen Anaplasma phagocytophilum
Source: mBio. 2024 Feb 28;15(4):e00299-24. doi: 10.1128/mbio.00299-24 (PMC11005342; doi:10.1128/mbio.00299-24)

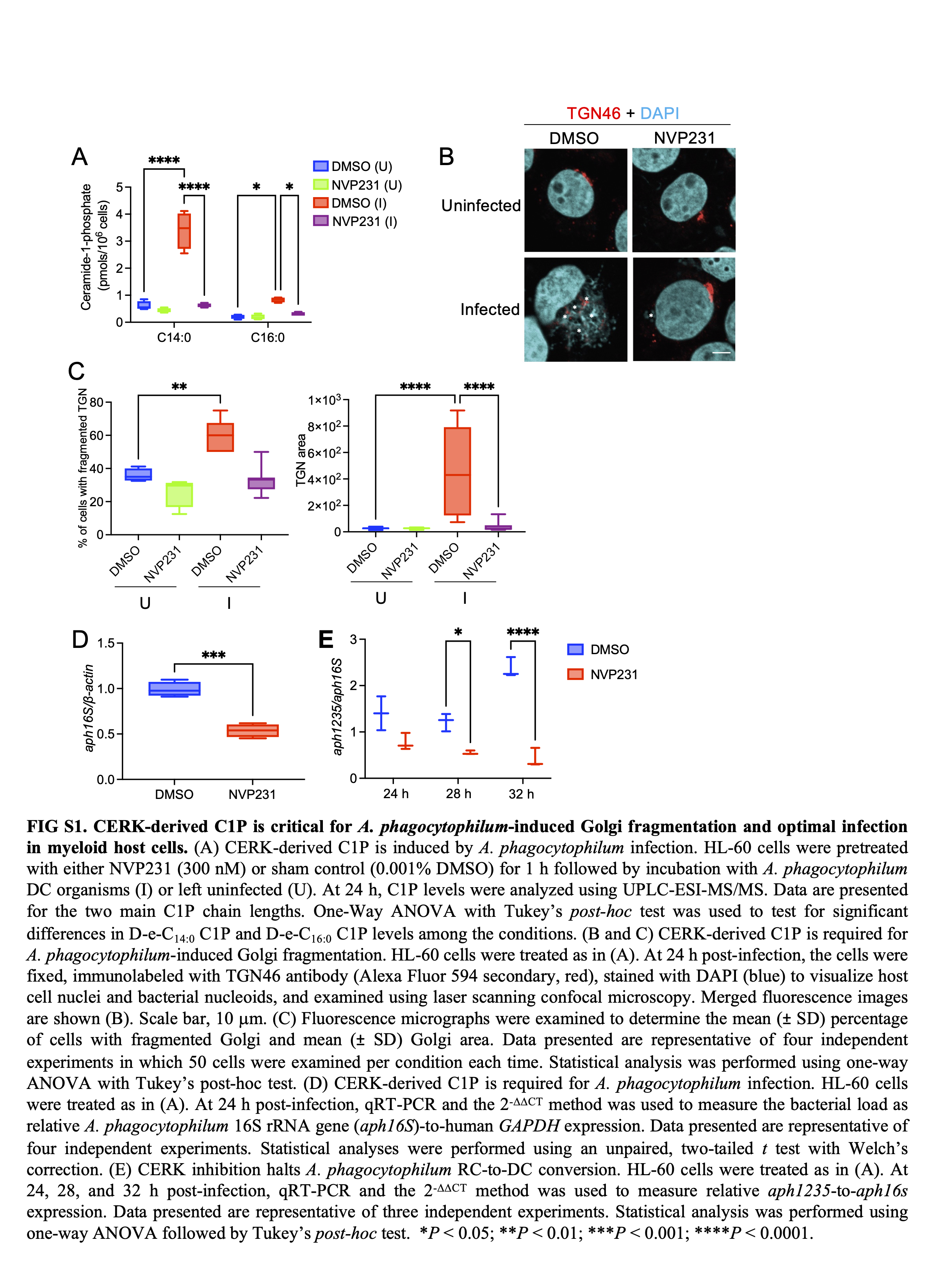

Supplement: Figure S1 — CERK-derived C1P is critical for A. phagocytophilum-induced Golgi fragmentation and optimal infection in myeloid host cells. [file mbio.00299-24-s0001.tiff]

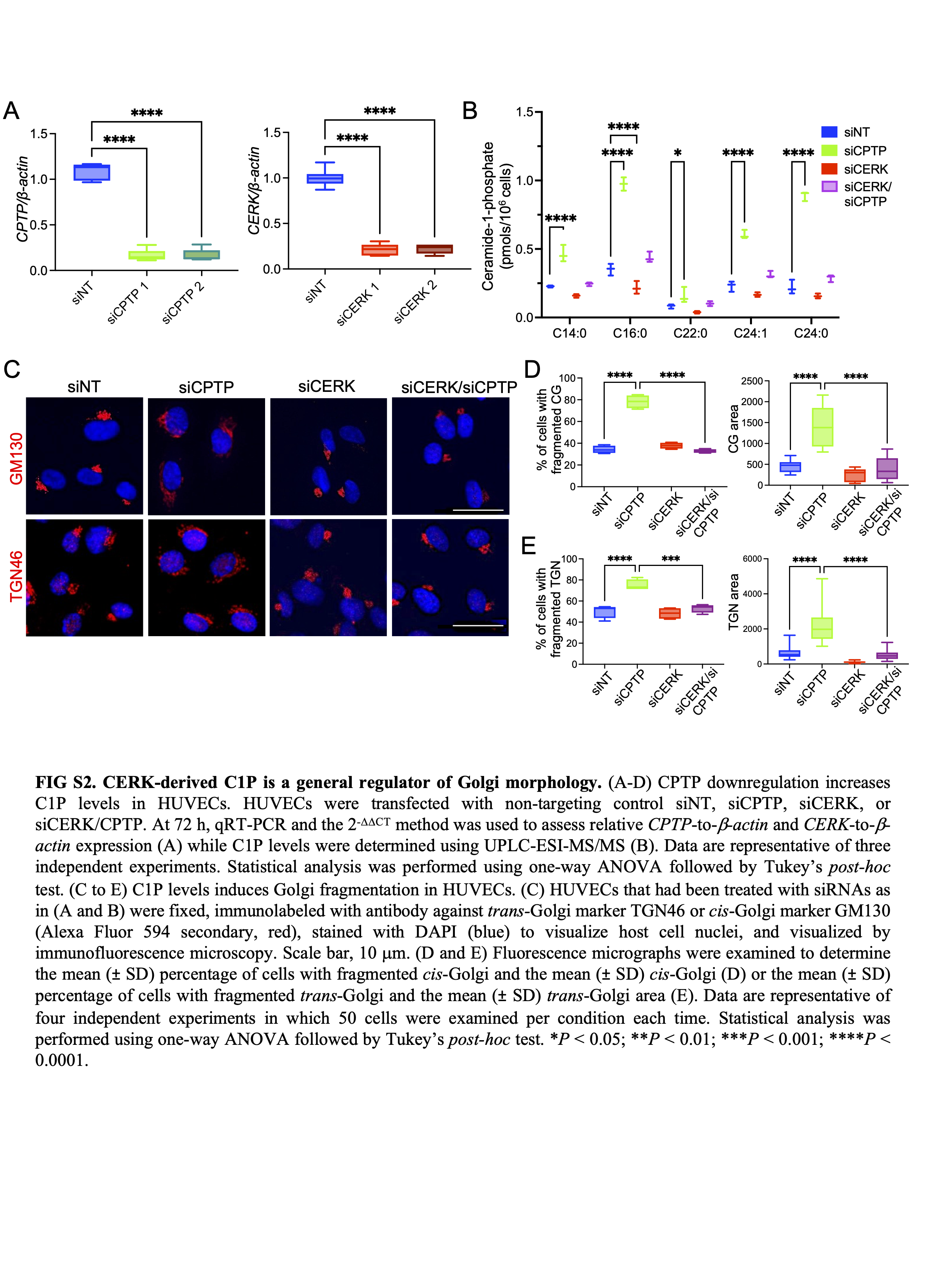

Supplement: Figure S2 — CERK-derived C1P is a general regulator of Golgi morphology. [file mbio.00299-24-s0002.tiff]

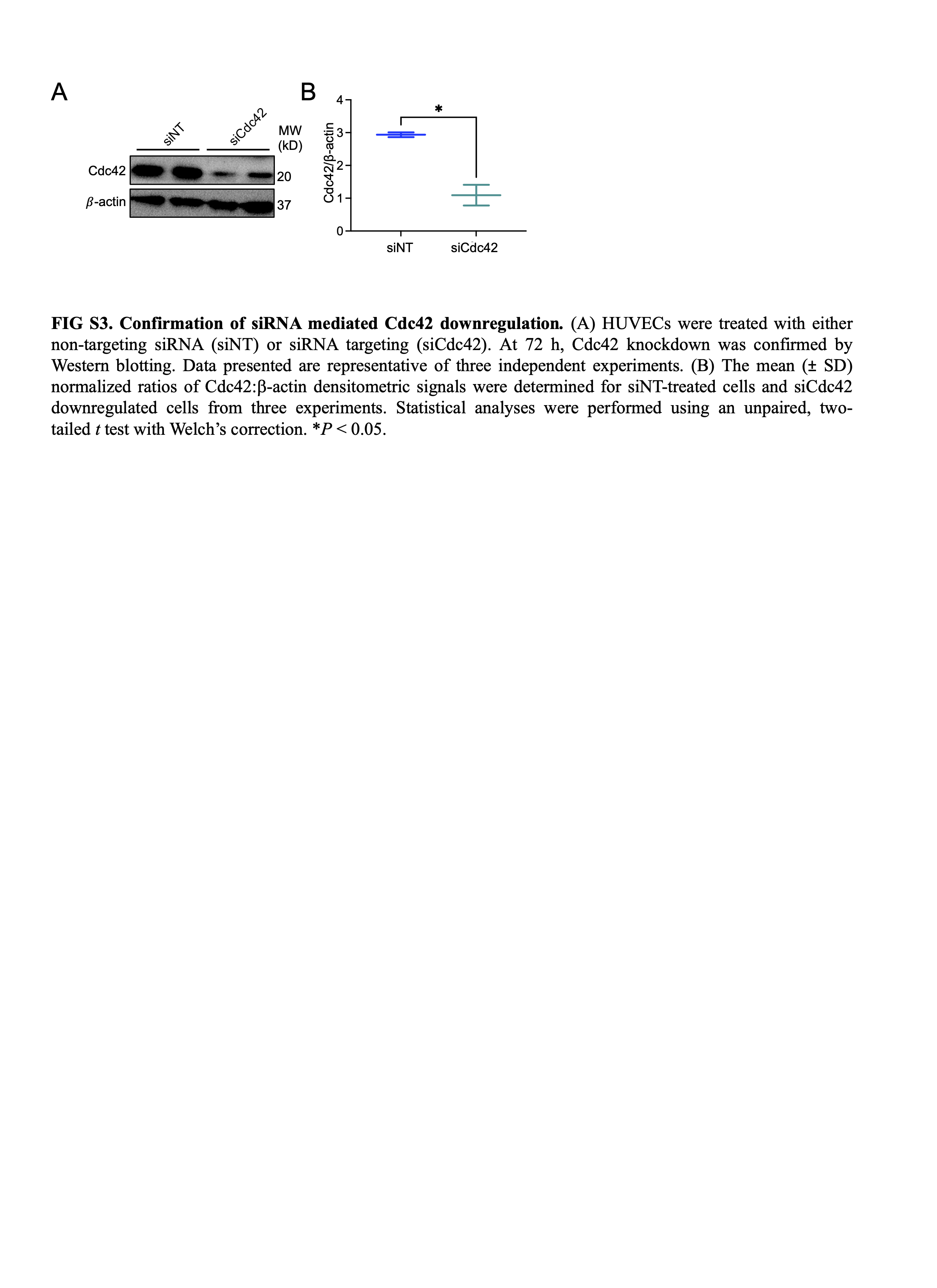

Supplement: Figure S3 — Confirmation of siRNA mediated Cdc42 downregulation. [file mbio.00299-24-s0003.tiff]
